# Visualising lignin quantitatively in plant cell walls by micro-Raman spectroscopy

**DOI:** 10.1039/d1ra01825f

**Published:** 2021-04-07

**Authors:** Xun Zhang

**Affiliations:** Beijing Key Laboratory of Lignocellulosic Chemistry, Beijing Forestry University Beijing 100083 P. R. China zhangxunyy@bjfu.edu.cn; National Forestry and Grassland Administration Key Laboratory of Plant Fiber Functional Materials Fuzhou 350002 P. R. China; Qilu University of Technology, Shandong Academy of Sciences, State Key Laboratory of Biobased Material and Green Papermaking Jinan 250353 P. R. China

## Abstract

As a main component in plant cell wall, lignin is commonly determined by wet chemical analysis which only provides general information rather than specifics for different cell wall layers. To address this issue, we attempted to use micro-Raman spectroscopy for quantitative visualisation of the lignin in various cell wall layers during delignification.

## Abstract 2

Lignin content in different cell wall layers was determined by micro-Raman spectroscopy.

## Graphical abstract

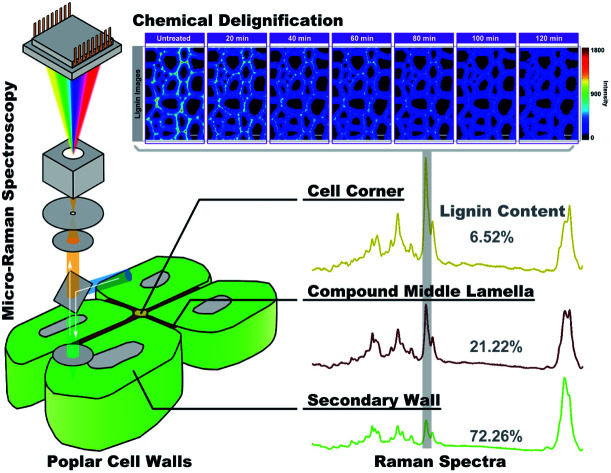

## Introduction

Plants have evolved to resist degradation and to confer robustness to the cell walls which are complex multiphasic structures with different compositions ordered on a number of scales from nanometres up to micrometres.^1^ This robustness or “recalcitrance” is attributable to its architecture rigidity and complexity *via* the spatial interaction of chemical compositions, especially the lignin.^2^ Because lignin has a high molecular weight and cross-links with other wall components, it minimises the accessibility of polysaccharides (including cellulose and hemicelluloses) to enzymes in biorefineries and results in protecting the cell walls against degradation.^3^ Therefore, lignin should be removed before breaking polysaccharides down into simple monosaccharides. For improvement of overcoming such recalcitrance, it is critical to quantitatively study on the lignin in plant cell walls, as well as the relationship among lignin, cellulose and hemicelluloses. The related research results are of great significance to pulp and paper making, biomass pretreatment, high value utilization of plant fiber, *etc.*

Conventionally, lignin content or concentration of plant cell walls is characterised by wet chemical analysis, which only provides average compositional data and is unable to trace the data of plant cell walls at a micro-level. Little knowledge of lignin content in different cell wall layers is available. In this regard, chemical imaging techniques can supplement results from macroscale testing. But the present techniques are illustrations showing qualitative images rather than quantitative data. Additionally, conclusions are sometimes inconclusive and, in part, contradictory, since powders are used for wet chemical analysis while sections are used for microscopic approaches.^4^

In this work, we attempted to quantitatively visualise the lignin of poplar cell walls by micro-Raman spectroscopy during delignification. Transverse sections of poplar xylem were applied for all the experiments to ensure the results of measurements from wet chemical analysis and microscopy were consistent or corroborate each other. Micro-Raman spectroscopy is a label-free and non-invasive tool for local detection of lignin in plants.^5^ By sampling and analysing tissues at varying periods of delignification, it offers an opportunity to trace the changes of lignin in plant cell walls.^6^ The delignification and Raman investigation are two independent operations, and therefore it is difficult to continuously monitor the changes within a fixed area of the sample. The spectra collected at different time are incomparable. Although quantitative information of each measurement is theoretically available by analysis of Raman spectra, the details of plant cell walls delignification at a micro-level is difficult to be described.^7^ To address this issue, the Raman signal of polysaccharides was taken as an internal standard for data calibration. The lignin content in different cell wall layers were displayed in a fixed area so that the spectra obtained at various time were comparable. Accordingly, the quantitative data of delignification within plant cell walls were achieved by further multivariate analysis.

## Materials and methods

### Sample preparation

A 10 year-old poplar tree (*Populus nigra* L.) was provided by Beijing Forestry University, China. Small sample blocks were cut out from the xylem. Without any embedding routing, 14 μm-thick transverse sections were prepared on a sliding microtome (Leica 2010R). Sections were then immersed in toluene/ethanol (2 : 1, v/v) at room temperature for 24 h to remove the extractives and dried in a vacuum oven at 50 °C for 12 h. Sections (1 g) were placed in a 250 mL conical flask containing 65 mL deionized water with 0.6 g sodium chlorite and 0.5 mL acetic acid, and heated in a water bath at 75 °C for 120 min to remove the lignin selectively. The delignified samples were collected by filtration at each time point (20, 40, 60, 80, 100, 120 min) for analysis of lignin content.

### Chemical compositions analysis

The chemical compositions analysis of the extractive-free samples was performed by using biomass compositional analysis laboratory procedures developed by National Renewable Energy Laboratory (NREL). The samples were hydrolysed with 72% sulfuric acid during 1 h at 30 ± 3 °C in a water bath. After dilution, hydrolysis was performed in an autoclave (Yxq-Ls-50SII, Yuntai) for 1 h at 121 ± 3 °C. Acid insoluble lignin was determined after filtration and hot water washing over a G4 glass filter crucible. Acid soluble lignin was determined by a UV spectrophotometer (UV2300, Techcomp) with 240 nm light. The supernatant fluid was diluted 15 times to bring the absorbance into the rage of 0.7–1.0. The monomer sugars were analysed by high-performance anion exchange chromatography (HPAEC) system (Dionex ICS 3000) with pulsed amperometric detector, AS50 auto sampler, CarbopacTM PA-20 column (4 × 250 mm, Dionex) and guard PA-20 column (3 × 30 mm, Dionex). Each sample should be analysed in duplicate.

The lignin content and polysaccharide content used in this paper are relative contents which are calculated by comparing absolute content before and after treatment. The absolute content of lignin is equal to the sum of acid soluble mass and acid-insoluble lignin mass divided by the dry mass of sample. The absolute content of polysacchrides is equal to glucose mass and xylose mass divided by the dry mass of sample.

### Micro-Raman spectroscopy

Section was placed on a glass slide with a drop of deionized water, and covered by a coverslip for subsequent micro-Raman investigation. Raman imaging data were acquired with a LabRam Xplora confocal Raman microscope (Horiba Jobin Yvon) equipped with a confocal microscope (Olympus BX51) and a motorized stage. Measurements were conducted with a high numerical aperture (NA) microscope objective from Olympus (60×, oil, NA = 1.35) to achieve high spatial resolution. A linear polarized laser (*λ* = 532 nm) excitation was focused with a diffraction-limited spot size (theoretical 1.22*λ*/NA). The Raman scattering was detected by an air-cooled front-illuminated spectroscopic charge-coupled device (CCD) behind a grating spectrometer (1200 grooves mm^−1^). For mapping, the step was set at 0.8 μm and every pixel corresponds to one scan. Spectrum of each location was obtained by averaging 2 s. Here, the mapping images are obtained by the peak height of acquired spectra.

### Data processing

Labspec5 software (Horiba Jobin Yvon) was employed to setup and control the microscope. The .ngc files, which are the standard format of the original imaging data, were converted to .mat files for further data analysis. The mathematical software MatlabTM R2014a (MathWorks) was employed to process the data. The original spectra were pre-processed to eliminate the spectral contaminants including baseline drifts and cosmic spikes before other data processing (*e.g.* principal component analysis and clustering analysis) are implemented.

## Results and discussion

The chemical compositions of untreated and delignified poplar samples are given in Table 1. A complete characterisation of untreated sample revealed that lignin accounted for 28.58% and polysaccharides accounted for 64.33% of the dry sample mass. The greatest removal of lignin occurred within the first 80 min of delignification, in which the lignin content was reduced from 100% to 70%. Only about 2% of original lignin was removed at the end of the treatment. The lignin content was 68% after 120 min of treatment. The polysaccharides content increased from 64% to 68% in the first 80 min of the treatment, and then decreased to 66%. Mass balance analysis shows that the polysaccharides content reduced from 100% to 93%, indicating that polysaccharides were also solubilised. This result is in agreement with the previous work of Siqueira *et al.*, who also observed that lignin removal is accompanied by a loss of polysaccharides during the chlorite treatment.^8^ Both lignin and polysaccharides decreased in the treatment, but lignin declined much more than polysaccharides. We suppose that the polysaccharides are difficult to extract from plants because of the protective action of lignin. Lignin removal is a crucial step before exposure of the polysaccharides fractions.

**Table 1:** Chemical compositions of untreated and delignified samples

Samples	Lignin content (%)	Polysaccharides content (%)
Untreated	100.00	100.00
20 min	93.77	98.74
40 min	79.88	97.72
60 min	78.55	97.54
80 min	70.47	97.40
100 min	69.74	94.92
120 min	68.73	93.40

**Fig. 1:**
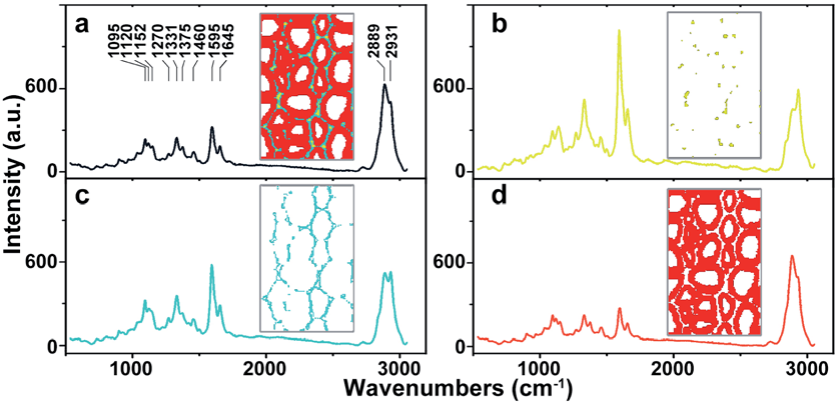
Average Raman spectra of (a) whole poplar cell walls, (b) cell corner (CC), (c) compound middle lamella (CML), (d) secondary wall (SW).

As a powerful tool for observation of vibrational, rotational and other low-frequency modes in a system, Raman spectroscopy is commonly used in wood chemistry to probe the relationship between structure, dynamics and function of biomolecules.^9^ Several organic compounds and functional groups can be identified by their unique spectral pattern. A typical Raman spectrum of untreated poplar is shown in Fig. 1a. Raman bands are attributable primarily to the major wood polymers including lignin and polysaccharides, and their assignments are displayed in Table 2 according to the previous literature.^10–12^ As a result of aromatic ring symmetric stretching vibration, typical bands of lignin appeared in the region between 1500 and 1700 cm^−1^. Here, the evident peak at 1595 cm^−1^ was employed to characterise the lignin in poplar. The bands at 1095 and 2889 cm^−1^ were attributed to C–O–C linkages and C–H stretching of polysaccharides, respectively. In particular, because the intensity of the peak located at 1095 cm^−1^ was sensitive to the orientation of the fibres with respect to the polarization direction, the peak at 2889 cm^−1^ was used to explore the chemical information about polysaccharides.^13^

**Table 2:** Raman peak positions and bands assignments for major structures of poplar

Wavenumbers (cm^−1^)	Components	Assignments
1095	C^a^, H^b^	Heavy atom (CC and CO) stretching
1120	C, H	Heavy atom (CC and CO) stretching
1152	C	Heavy atom (CC and CO) str. plus HCC and HCO bending
1270	L^c^	Aryl-O of aryl OH and aryl O–CH_3_; guaiacyl ring (with C=O group) mode
1331	L, C	HCC and HCO bending
1375	C	HCC, HCO, and HOC bending
1460	L, C	HCH and HOC bending
1595	L	Aryl ring stretching, symmetric vibrating
1654	L	Ring conjugated C=C str. of coniferyl alcohol; C=O stretching of coniferaldehyde
2889	C, H	CH and CH_2_ stretching
2931	L, C, H	CH str. in OCH_3_ asymmetric vibrating

a Cellulose.

b Hemicelluloses.

c Lignin.

The native poplar cell walls are organized in several layers that consist of the cell corner (CC), compound middle lamella (CML, middle lamella plus adjacent primary wall), and secondary wall (SW, with S1, S2 and S3 layers).^14^ By using principal component analysis (PCA) and clustering analysis (CA), poplar spectra (removal of the spectra of cell lumen) were extracted and classified into three groups according to their features, *i.e.* CC, CML and SW.^15^ Their average spectra are shown in Fig. 1b–d, respectively. Results indicate that the spectra of various cell wall layers have the same peak positions but different intensities. The lignin peak is more pronounced in the average spectra of CC and CML than those in SW. The polysaccharides are opposite to that of lignin; the SW has higher intensity than CML and CC.

**Fig. 2:**
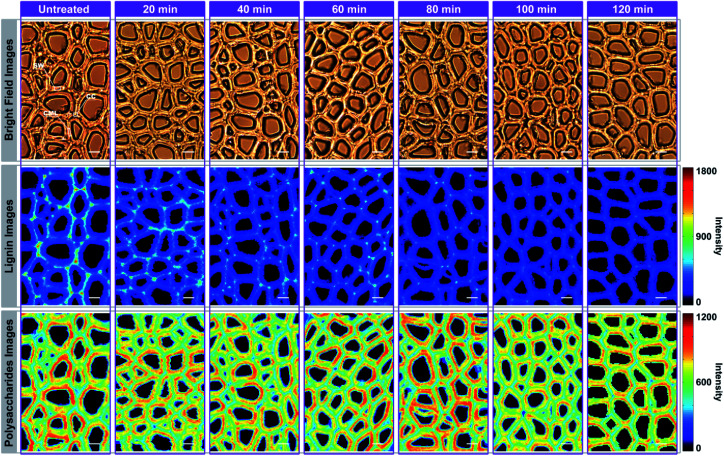
Raman imaging of poplar cell wall at specific time during delignification: bright field images of poplar cross-section (first row); lignin images (integrating over the 1595 cm^−1^ band) (second row); polysaccharides images (integrating over the 2889 cm^−1^ band) (third row). Bar = 8 μm.

**Fig. 3:**
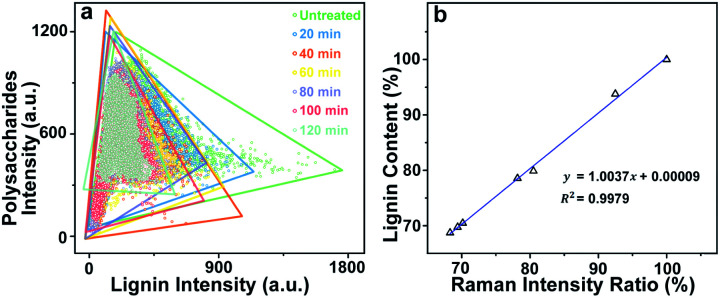
(a) The plots of lignin and polysaccharides Raman signals (1595 cm^−1^*vs.* 2889 cm^−1^) at different treatment time, (b) calibration curve produced by intensity ratios and chemical compositions analysis.

For Raman imaging, the bright field images were used to record the spatial information of the untreated and delignified poplar, and spectra were measured over a 106.4 μm × 68.8 μm region with a spatial resolution 0.8 μm per pixel. Consequently, 11 438 spectra were obtained from each sample and can be regarded as a matrix of 11 438 by 977 dimensions (each spectrum consists of 977 data points). Bright field images and their corresponding Raman images achieved by integrating specific Raman peaks are presented in Fig. 2. The bright field images unveil that the poplar fibres were still complete after delignification. No significant morphological change was observed implying that the delignification did not destroy the network structure. To analyse in details the lignin and polysaccharides changes during the delignification, the plots of their Raman signals (1595 cm^−1^*vs.* 2889 cm^−1^) at different treatment time are shown in Fig. 3a where the characterisation of each spectrum can be easily distinguished. The data points in the diagram were distributed mostly in a triangle area. In the Raman images, the bright regions represent high concentrations of specific chemical compositions, while the dark regions represent low concentrations. Due to the dissolution of lignin and polysaccharides, intensities of their Raman images show a tendency to decrease with increasing time of the treatment. The reductions of lignin signal intensity in the CC and CML are more important than that in SW. In contrast, the entire signal intensities of polysaccharides decrease almost simultaneously, indicating that there is no marked preferential removal of polysaccharides at different cell wall layers.

Although the chemical changes are visualised by using micro-Raman spectroscopy, the Raman images only provide qualitative information. To quantitatively explore the poplar cell walls delignification by using Raman imaging data, we have to consider two issues: (1) the comparability of Raman data obtained at different treatment time, and (2) the relationship between the Raman intensities and chemical compositions analysis. Previous results revealed that the poplar cell walls and the polysaccharides distribution remained their original features after the treatment. Hence, the bright field image of untreated sample can be regarded as a template for describing the delignification process. Based on the polysaccharides signals (*i.e.* the internal standard), the template was then filled with the spectra collected at various treatment times so that the dynamic changes of lignin during delignification were displayed in a fixed area.

A Raman imaging data was a matrix **X** of dimension *m* by *n*, in which the digitized Raman spectrum of each scanning corresponded to a row vector in the data table:1
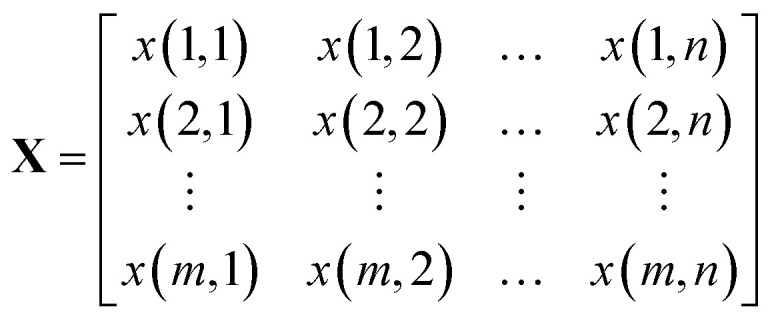
where *m* is the number of spectra traces in this dataset and *n* is the number of data points per spectrum along the wavenumber (or any other spectral variable) axis. Specifically, the polysaccharides intensities (2867 to 2920 cm^−1^) of untreated sample and treated sample were extracted and written as matrices **X**_un_ and **X**_tr_ with dimension *m* by *l*, respectively. They were normalized as:2
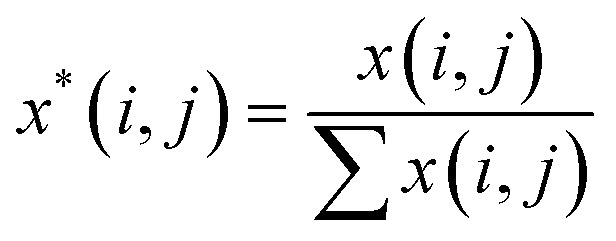


The squared Euclidean distance array *D*(*p*,*q*) between two spectra was calculated as:3
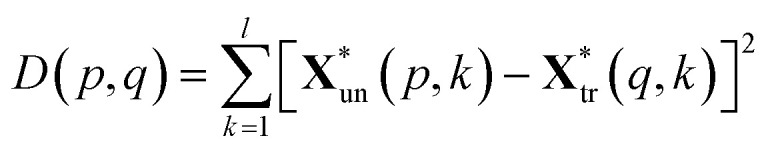
where 
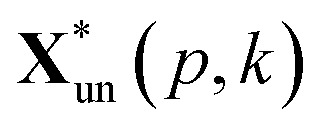
 and 
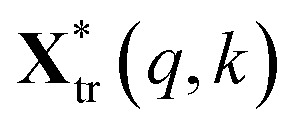
 are the *p*th and *q*th spectrum in polysaccharides signals. The filled spectrum was determined by finding the minimal Euclidean distance between the polysaccharides features of untreated and treated samples. The data of treated poplar cell walls were presented in a fixed area so that the spectra obtained at different time are comparable. Raman intensity ratio after treatment was calculated as described in eqn (4).4
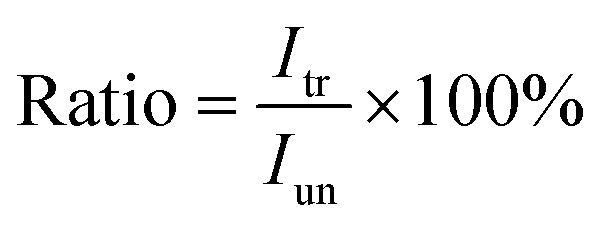
where *I*_un_ and *I*_tr_ are the accumulation of lignin intensities in spectra of untreated sample and filled spectra of treated sample, respectively. The ratios and the lignin content (acquired by chemical compositions analysis) were combined to produce the calibration curve, which is linear thereby obeying the Lambert–Beer law (Fig. 3b). The regression slope was 1.0037 and the coefficient of determination (*R*^2^) was 0.9979 indicating that the ratios can be employed to describe the remaining lignin content quantitatively.

**Fig. 4:**
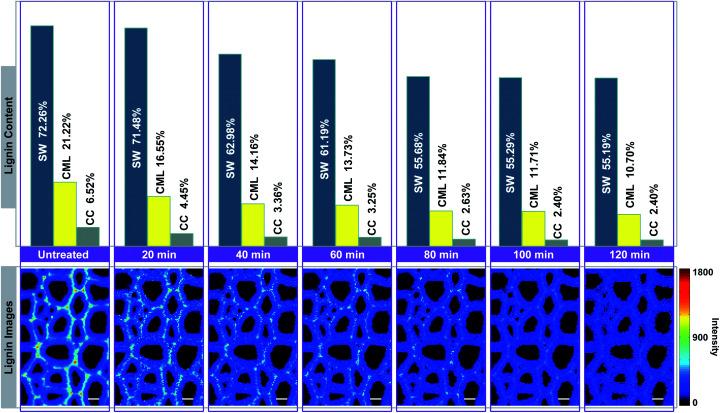
Simulation of lignin Raman images for visualising the delignification of poplar cell walls. Bar = 8 μm.

In Fig. 4, the Raman images of lignin are simulated by using the peak at 1595 cm^−1^ of the calibrated Raman spectra, and the histograms (quantitative data) were constructed by calculating the ratios between the intensities of each cell wall layer and untreated sample. The untreated sample has a heterogeneous distribution of lignin within various cell wall layers, clearly displaying high contents in CC, followed by CML, and lowest in SW. However, the largest amount of lignin is found in the SW (72.26%), while CML and CC only account for 21.22% and 6.52%, respectively. This is because that the SW occupies most of the cell wall mass in the poplar xylem.^16^ The lignin content of poplar sample decreased with treatment time in all cell wall layers, but the delignification process in different regions were independent.

The lignin content of SW undergoes two rapid drops occurred at periods of 20–40 min and 60–80 min, in which 8.50% and 5.51% of lignin were removed, respectively. It remained fairly constant in the first 20 min (only 0.78% of lignin is solubilized). The same situations were also found in the subsequent periods of observation (40–60 min and 80–120 min). For CML, it was found that a large amount of lignin was solubilized in the first 40 min (7.06%), 60–80 min (1.89%) and 100–120 min (1.01%) of the treatment. In contrast, the lignin content decreased slightly at periods of 40–60 min and 80–100 min. The initial removal of lignin in the CC was similar to that of the CML, where the lignin was quickly solubilized in the first 40 min. The remaining content of lignin, however, was tending to be constant from 40 min to the end of the treatment. The results indicated that the preferential distinctions existed among the delignification of various cell wall layers.

It seems that the lignin in the CC is much easier to be removed than that in the SW, which may be ascribe to the differences of the structure in these two layers. Specifically, delignification of the SW can be divided in to two stages: (1) slow penetration of reaction liquid, (2) followed by rapid removal of lignin. CML shows a transient state between CC and SW. The compact deposition of polysaccharides is likely to impede the penetration of reaction liquid into the cell wall layers, especially the SW with highest polysaccharides content. In addition, the CC regions contain mostly guaiacyl residues (G-type lignin) while syringyl residues (S-type lignin) are predominant in the SW.^17^ We suppose that the G-type lignin is preferentially dissolved in the solvent during the delignification. But such hypothesis remains to be confirmed by future studies.

## Conclusion

Overall, the lignin of poplar cell walls was *in situ* visualised by applying micro-Raman spectroscopy. A linear relationship between Raman intensities and chemical compositions was used as the basis for the quantitative illustration of lignin removal in different cell wall layers. We established a linkage between the micro-level behaviours and the macro-level performance of plant cell walls before, during, and after delignification. This linkage would serve to greatly increase the mechanistic understanding of delignification, and provide a potential way to understand the cell wall deconstruction in biorefineries.

## Conflicts of interest

There are no conflicts to declare.

## Supplementary Material
